# Social Memory in the Mekong’s Changing Floodscapes: Narratives of Agrarian Communities’ Adaptation

**DOI:** 10.1007/s10745-022-00362-0

**Published:** 2022-10-04

**Authors:** Thong Anh Tran, Jonathan Rigg, David Taylor, Michelle Ann Miller, Jamie Pittock, Phong Thanh Le

**Affiliations:** 1 Asia Research Institute, National University of Singapore, Singapore, Singapore; 2 Fenner School of Environment and Society, College of Science, The Australian National University, Canberra, Australia; 3 School of Geography, Earth and Atmospheric Sciences, Faculty of Science, The University of Melbourne, Victoria, Australia; 4 Fulbright School of Public Policy and Management, Fulbright University Vietnam, Ho Chi Minh City, Vietnam; 5 School of Geographical Sciences, University of Bristol, Bristol, UK; 6 Department of Geography, National University of Singapore, Singapore, Singapore; 7 Climate Change Institute, An Giang University, VNU-HCM, Long Xuyen City, An Giang Vietnam

**Keywords:** Community resilience, Human–environment interactions, Social memory, Transformative adaptation, Vietnamese Mekong Delta

## Abstract

Rural adaptation encompasses place-based perceptions, behaviors, livelihoods, and traditional ways of life associated with local environments. These perceptions, norms, and practices are disturbed by coupled environment-development externalities. This study employs the Vietnamese Mekong floodplains as an exemplary case to illustrate how floods impact agrarian communities and how they have experienced flood alterations driven by hydropower development and climate change in recent years. Drawing on thematic and narrative analyses of qualitative data (focus group discussions and interviews) collected in three agrarian communities in the Vietnamese Mekong floodplains, sources drawn from various news outlets, and academic materials, we argue that disrupted flood environments in the floodplains have triggered affective flood reminiscences, catalysing shifts to incremental and transformative adaptation to achieve resilience. We build a nuanced understanding of how social memory helps to enhance human–environment relationships in response to highly complex hydrological dynamics in the delta.

## Introduction

State policies play a crucial role in shaping development-environment relationships. In Southeast Asia, while adverse impacts of climate change are evident, large-scale development projects such as hydropower dams led by governments and corporations have disturbed human-resource dependencies and created new human-nature relationships (Rigg et al., 2016; Hecht et al., 2019; Kenney-Lazar, 2019). In the Vietnamese Mekong Delta (VMD), the legacies of the former colonial (French) administration present challenges for current decision-makers to resolve technical and historical issues associated with flood management and related environmental issues (Biggs, 2011). Since the Renovation period (*Đổi Mới)* in the late 1980s, which marked a significant shift towards a market-based economy, the central government has focused on exploiting the VMD for economic development. Large and thinly populated wetland areas have been converted into highly-regulated agricultural landscapes, supported by water-engineering systems to control floods and regulate irrigation for rice production (Nguyen et al., 2016). Creating these engineered landscapes involves restructuring technical, economic, and human resources and reorganising adaptation activities to accommodate emerging environmental challenges in the floodplains.

These development processes, taken together, have not only boosted rice production but have also ushered in complex, far-reaching, and enduring environmental problems. In the VMD, intensive farming practices (using heavy machinery, chemical pesticides, and fertilizers) coupled with the transboundary effects (reduced sediment and nutrient flows) of large hydropower dams located upstream and climate variability (El Niño effects) have severed human-resource connections and degraded floodplain ecosystems (Hoang et al., 2019). Although recent studies of the VMD floodplains have attached greater attention to local impacts of infrastructure systems (e.g., roads and dykes) (Dang et al., 2016), the role of place-based perceptions of transboundary impacts associated with hydropower development and climate change in stimulating transformative adaptation has been under-explored. We investigate how local agrarian communities view and articulate these externalities as they navigate rupture processes and accelerate disconnection to floods in their everyday lives.

Social memory is defined as accumulated experiences and collective history of a given community in managing resources, making sense of place-based environments, and adapting systems to social-ecological change (Mistry et al., 2014; Nykvist & von Heland, 2014; Valencia et al., 2019). We employ this concept to illustrate how these human–environment connections have changed as riparian communities in the VMD have adapted to increasingly unpredictable and intense variations in “living with floods” and “living without floods.” While riparian communities comprise heterogeneous actors with different individual perspectives and worldviews, specific shared experiences can produce corresponding shifts in collective environmental behaviors and associated livelihoods. Adopting the “living with floods” perspective long practised by such floodplain communities (Table 1), we use social memory as an entry point to analyse how environmental impacts are retrospectively linked to drivers and processes of change, informing corresponding changes in adaptive behavior and action. Social memory is a valuable heuristic that helps analyse how agrarian communities collectively appreciate the environmental value of seasonal floods and interpret changing floodscapes to trigger behavioral shifts that enhance collective resilience (Wilson, 2015). While the “living with floods” narrative has been interpreted and applied quite extensively (albeit differently) in formal policy documents and empirical studies (Danh & Mushtaq, 2011; Liao, 2019), the social-psychological processes behind transformative behaviors in agrarian societies from a social memory perspective are, by comparison, relatively understudied.

**Table 1:** Terms associated with floods used in the study

Term	Interpretations
Living with floods	(1) As a community-driven concept, it refers to a traditional adaptive strategy adopted by rural flood-prone communities in the VMD. It is premised on their full realisation of the non-preventive nature of floods while realising their negative and positive implications for local socio-economic development and the lives of rural communities (Dang & Pham, 2003) (2) A government-driven economic approach refers to controlling floods for growth, often through the displacement and resettlement of landless farmers and fishers (Liao et al., 2019)
Too much/too little floodwater	Unpredictable conditions of water systems in the delta are associated with compounding transboundary and in-situ impacts of hydropower dams and climate change
Beautiful floods	An aesthetic term referring to the “acceptable” intensity and magnitude of floods commonly perceived by rural communities (Ehlert, 2012), usually associated with flood peaks of about 4.0–4.5 m (Le et al., 2007)
Flood reminiscence	Social memories of flood seasons and past forms of community engagement with floods
Flood-longing	An expression of yearning for the arrival of (usually overdue) flood seasons (Le, 2019)
Flood season full of sadness	Emotional stress is felt by agrarian communities on delayed flood arrival (Quoc et al., 2019)
Flood starvation	An expression used to convey feelings of extreme deprivation from, and hunger for, floods in the context of overall declining flood flows (Cuu, 2019)

In exploring social memory, we examine the specific phenomenon of “flood starvation,” the collective perceptions and experiences of extremely low flood conditions that adversely impact resource-dependent livelihoods and ways of life in the VMD floodplains (Luc, 2019). Here, flood starvation denotes the idea – broadly agreed upon by agrarian residents in a given community – that floodwaters are unusually low to the point that they cannot provide essential ecosystem services for livelihoods and ways of life. Grounded on qualitative data gathered from focus group discussions (FGDs) and interviews with key informants in three flood-prone and, therefore also, potentially, flood-starved areas (An Giang and Dong Thap Provinces and Can Tho City), we argue that disrupted flood environments in the VMD floodplains have triggered affective flood reminiscences among agrarian communities, catalysing shifts in adaptation practices (incremental and transformative) aimed at building and enhancing resilience. We use “incremental” and “transformative” adaptation to refer to two distinct but interrelated and sometimes overlapping change processes. While incremental adaptation is gradual, distributed, often locally distinct, and community-based, transformative adaptation tends to be spatially dispersed and driven mainly by external events (Wilson et al., 2020). In practice, these two processes often intersect and occur concurrently.

## Conceptual Framework

### Social Memory in Changing Floodscapes

Social memory originates from individuals and institutions and draws on many practices, knowledge, and values to prepare systems for change, build resilience, and cope with surprises (McEwen et al., 2016). As Drozdzewski et al. (2016) claim, it is a powerful force that invokes collectivised experiences, emotions, and an awakening of the senses to deal with change. Social memory creates linkages between the past, present, and future, which shift over time through interwoven processes of social learning, experimentation, experience, and innovation shaped by situated activities or events (Dickson-Hoyle et al., 2020; Reid et al., 2018). Contextualised in the coupled human-flood systems of the VMD, social memory serves as a thread connecting collective memories of agrarian communities of changing floods to their knowledge, experiences, and emotions – hence provoking affective flood reminiscences that inform differential typologies of adaptation.

Numerous studies have addressed the role social memory plays in building community resilience in response to environmental challenges (Mistry et al., 2014; Wilson, 2015; McEwen et al., 2016; Garde-Hansen et al., 2017; Valencia et al., 2019). Exhibited in various forms of human behavior, social memory serves as a collectively shared mental map that enables communities to experientially develop resilience capacities to tackle negative externalities (Barthel et al., 2010; Folke, 2006). In the context of flood risk management, flood memory provides a learning platform through which to develop and disseminate knowledge and create valued opportunities to increase collective resilience capacities (McEwen et al., 2016). How communities experience environmental threats and crises shapes how they can devise and adopt innovative livelihood strategies to navigate complex environmental challenges (Koczberski et al., 2018).

**Fig. 1:**
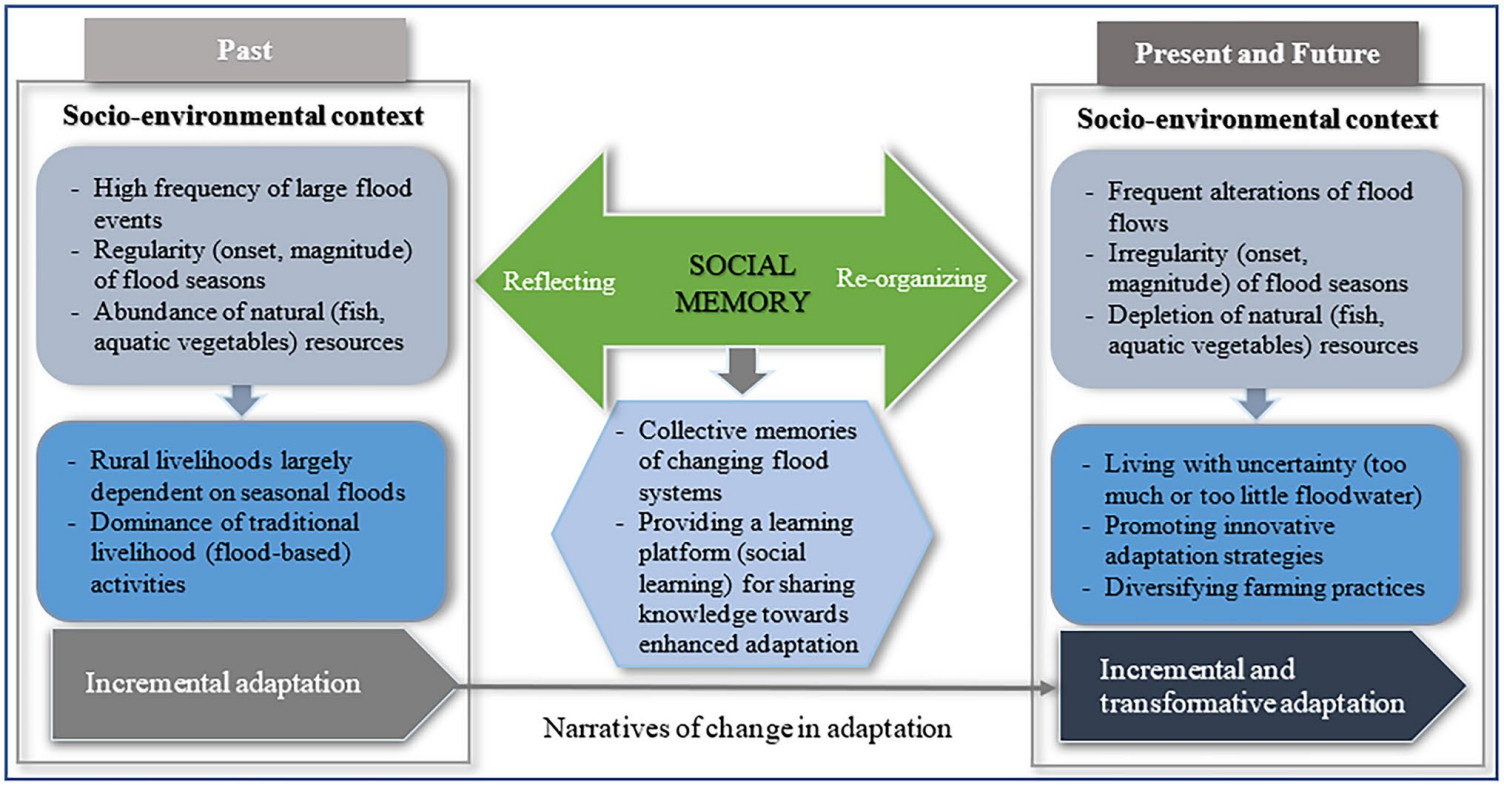
Social memory and narratives of adaptation in the VMD floodplains

Socio-cultural factors often determine adaptation (Curry et al., 2021; Nielsen & Reenberg, 2010). From the lens of social memory, it illustrates the ways agrarian communities reflect historical processes of flood systems they experienced in the past, whereby they learn and reorganise activities to adapt to emergent flood conditions at present and in the future (Fig. 1). Here, we see it as a learning thread that connects agrarian communities’ “looking-backward” experiences to “looking-forward” strategies that help them better adapt to flood challenges. Drawing on the idea of “remembering as resilience” described by Garde-Hansen et al. (2017), the study explores how social memory captures agrarian communities’ past experiences of environmental events that initiate various forms of incremental adaptation and how these catalyse their reorganisation of adaptive responses (including transformative adaptation) to enhance resilience. In this sense, a social memory perspective is essential to decipher real-life experiences, imaginaries, and adaptation strategies in response to largely unseen transboundary decision-making and development processes in the Mekong region. Human–environment systems may be coupled from an ecological standpoint, but, viewed from the perspective of riverine communities whose livelihoods rely upon agricultural and aquacultural production in the VMD floodplains, they appear to be disconnected. Transformative adaptation is therefore driven by the transboundary nature of systems but shaped, in its detail, by local experiences and imaginaries.

### Typologies of Adaptation and Shift towards Resilience

Environmental change prompts societies to deploy strategies to mitigate impacts or seize opportunities to overcome challenges. Two primary forms of adaptation – incremental and transformative – have gained traction in the literature on climate change adaptation (Fedele et al., 2019; Wilson et al., 2020). Employing the lens of social memory, this study will explore how these adaptation patterns evolve and shift over time, as exhibited through community narratives of living with floods in the VMD.

Climate change responses are commonly characterised as incremental adaptation, mainly operating over the short-term and at a local level and small-scale (Wilson et al., 2020) (Table 2). Given its narrow spatial–temporal scope in function and operation, this approach may fail to address the root causes of vulnerability (Fedele et al., 2019). In this study, incremental adaptation characterises small-scale adjustments in adaptation strategies undertaken by agrarian individuals/groups, such as shifting farming calendars to accommodate changing flood conditions.

**Table 2:** Dimensions of incremental *versus* transformative adaptation concerning evolutionary resilience

Dimensions of incremental *versus* transformative adaptation		Evolutionary resilience
Incremental adaptation	Transformative adaptation	
Decisions or behavioral changes took at the individual level	Collective actions contribute to changes in social systems and the natural environment Institutional reforms with government-led initiatives	“Bounce-forward” resilience Enabling reform
Changes at the local scale	Changes taking place at regional and broader scales	Emphasises transformation or path creation in response to disturbances (do something different)
Short-term processes	Long-term processes	Long-term response
Supports coping	Supports resilience	

Sources: Adapted from Scott (2013) and Wilson et al. (2020)

Transformative adaptation is needed to deal with environmental impacts when incremental adaptation alone is not sufficient (Kates et al., 2012). Such transformative adaptation is the most viable approach for long-term adaptation to a changing environment (Wilson et al., 2020). Moving beyond the operational capacities of incremental adaptation, transformative adaptation involves fundamental changes in institutional arrangements, priorities, and norms (Kates et al., 2012). Novel environmental challenges in the VMD induced by disruptive floodwater levels have prompted agrarian societies to adopt both incremental and transformative adaptation pathways. This is mainly characterised by community-driven efforts in developing innovative adaptation strategies to tackle emerging challenges (Rigg & Oven, 2015).

From the social-ecological perspective, it refers to the “capacity of a system to absorb disturbance and re-organise while undergoing change” (Berkes & Ross, 2013, p. 6). Existing literature presents three distinct perspectives of resilience, including engineering, ecological and evolutionary resilience. The broader literature on resilience involves critical debates on how resilience is conceptualised. While engineering resilience solely emphasises the ability of the system to absorb or accommodate disturbances without experiencing change (Holling, 1973), ecological resilience emphasises persistence, change, and unpredictability (Davoudi et al., 2013). Evolutionary resilience emphasises the “bouncing-forward” nature of the system. It emphasises active transformation, adaptation, and the search for – and formulation of – alternative development strategies (Davidson, 2010). Evolutionary resilience embraces typical attributes of transformative adaptation and provides an important method of analysis for rural studies (Scott, 2013).

Historical understandings of past environmental repercussions prompt agrarian societies to learn, reorganise, and develop various adaptation strategies to build resilience to future threats and perturbations (Bruijn et al., 2017). In this study, we use the concept of evolutionary resilience to demonstrate how agrarian communities, when stimulated by social memory, have shifted from incremental to transformative adaptation. Within the new challenging context of the VMD, evolutionary resilience is characterised by the self-organising capacity of agrarian communities through: (1) continuously learning to live with environmental change and uncertainty; (2) promoting innovative adaptation strategies; and (3) diversifying farming practices (Marschke & Berkes, 2006; Nguyen & James, 2013) (see Fig. 1).

This study contributes to a nuanced understanding of resilience operating at the community level in seeing social memory as a critical component of the everyday adaptation practices of agrarian communities. It serves as a learning thread allowing communities to make sense of changing flood regimes by connecting their past experiences of living with floods to inform current and future strategies to deal with change. Such evolving adaptation practices, reframed by the social memory of community-flood interactions over time, would create resilient communities that can confront extreme flood situations (too little or too much floodwater).

## Research Methods

### Selection of Case Studies

**Fig. 2:**
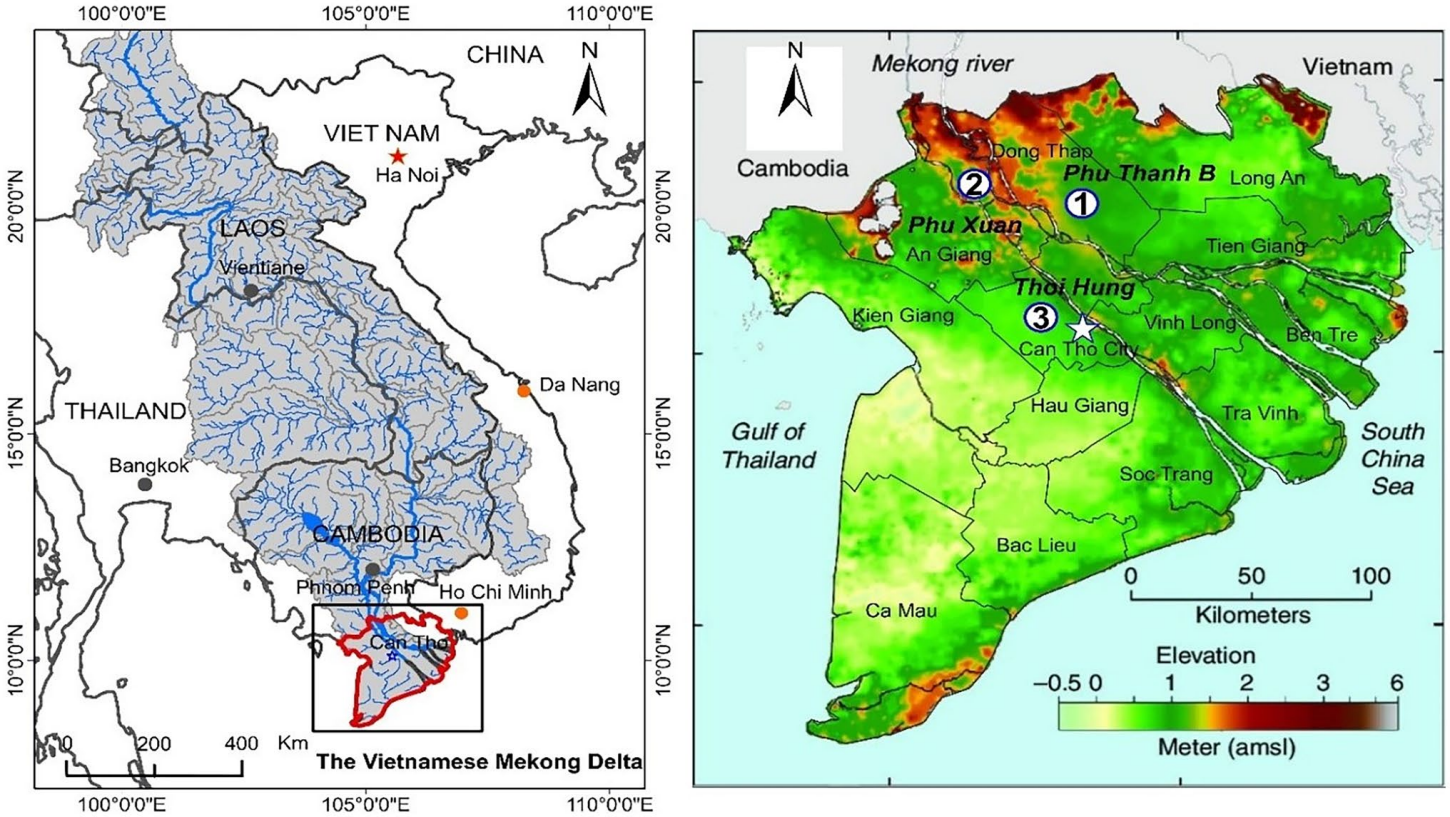
The maps of the Lower Mekong Basin (left) and the study areas in the VMD floodplains (right): (1) Phu Thanh B, (2) Phu Xuan, and (3) Thoi Hung. Source: Base map adapted from Minderhoud et al. (2019)

Three agrarian communities in the VMD with different flood control dykes were selected for this study: (1) Phu Thanh B, Tam Nong District, Dong Thap Province; (2) Phu Xuan, Phu Tan District, An Giang Province; and (3) Thoi Hung, Co Do District, Can Tho City (Fig. 2). Geographically, these study areas represent three distinct landform units in the delta and are characterised by differential structural systems, including the Plain of Reeds (Phu Thanh B – low dykes), the upper floodplain (Phu Xuan – the North Vam Nao flood control scheme), and the tide-affected floodplain (Thoi Hung – locally-designed high dykes) (Tanaka, 1995; see also Tran & James, 2017). While agriculture is the primary means of livelihood for local communities, collecting natural resources (fish, aquatic vegetables) or practicing integrated farming systems (rice-fish) also provides them with additional income during the monsoon season (starting in July and ending in November). These practices enable agrarian communities to observe and reflect a change in flood systems that directly impacts their livelihoods.

The rationale for selecting these study areas is that local agrarian communities have profound experiential knowledge of living with floods, forming an inherent part of their riverine lifestyles (Taylor, 2001). Long-term engagement with seasonal floods allows agrarian communities to accumulate flood memories and understand how flood changes affect their everyday lives and flood-based livelihoods.

### Data Collection and Analysis

An exploratory research approach was employed to examine how human-flood relationships in the VMD are (re)constituted through social memory. This approach directs attention to how agrarian communities make sense of altered flood regimes that affect their everyday lives and livelihoods and reimagine their futures correspondingly. The study entailed three stages of data collection. It started with nine FGDs and thirty-three interviews from September 2013 to March 2014 (the first author’s Ph.D. research). It was followed by twelve interviews between February and April 2019, three in November 2020, and three in September 2021 (Table 3). Given travel constraints due to the Covid-19 pandemic, interviews through Skype and phone calls were conducted in the last two rounds. While this timeframe is indeed very short to assess changes in perceptions about flood regimes, which, ideally, should be measured over several decades, it attests to the rapid pace at which transformations take place in the Mekong region. This also allows the author to follow up on changes in local flood systems, capturing the narratives of how the respondents experienced and adapted to the on-the-ground situations.

**Table 3:** Summary of focus group discussions and interviews for the study

Fieldwork activities	Qualitative methods	Participants	Approaches for data collection and analysis	Themes focused
The first round	Focus group discussions (nine FGDs)	Household groups (poor, medium, better-off) in three communes: Phu Thanh B, Phu Xuan, and Thoi Hung	Recruitment of participants for FGDs was based on the participatory approach (King & Horrocks, 2010; Neuman, 2011) Thematic and narrative analyses (Neuman, 2011) using NVivo software (Bazeley & Jackson, 2013)	Dyke development and impacts on livelihoods Changes in flood systems and critical drivers Change in farming practices at the community level
	Semi-structured interviews (thirty-three interviews)	Environmental scientists, agricultural experts, government officials across administrative levels, and farmers	Purposive and snowball sampling approaches (Liamputtong, 2013) Thematic and narrative analyses (Neuman, 2011) using NVivo software (Bazeley & Jackson, 2013)	Flood situations in the floodplains Drivers of hydrological change in the delta and corresponding impacts Adaptation practices at the institutional level Responses to environmental challenges in the future Agricultural systems in the new environmental context
The last three rounds	Semi-structured interviews (eighteen interviews)	Environmental scientists, agricultural experts, government officials at the national and provincial levels, and farmers	Purposive and snowball sampling approaches (Liamputtong, 2013) Thematic analysis (Neuman, 2011) using NVivo software (Bazeley & Jackson, 2013)	Emerging environmental conditions characterised by hydrological change under impacts of upstream hydropower dams, climate change, and dyke systems in the floodplains Incremental and transformative adaptation practices at the community level

Respondents from household groups (poor, medium, and better-off) and key informants were recruited for FGDs and interviews in the first stage of the study. The recruitment of the respondents was based on their long-term engagement with floods (through livelihood practices) and knowledge of local flood conditions. The key informants involved in the interviews comprised environmental scientists, agricultural experts, government officials, and senior farmers (i.e., farmers with prolonged experiences of living with floods). The selection of FGD participants was determined in collaboration with government officials at the study sites. Following Ellis and Freeman (2004), land ownership, income level, source of income, and housing conditions were factored into the selection process and used to categorize households as poor, medium wealth, and better-off. Poor households were defined as landless or land poor (< 0.5 ha), with their primary income derived from wage labor. Medium households often owned more extensive agricultural land (0.5–2 ha) and depended on the mix of on-farm and off-farm work. Better-off households often owned more than 2 ha and primarily engaged in farming. Lines of discussion relating to their experiences of flooding comprised: (1) changes in inter-annual flood cycles, (2) flood management policies, (3) locally-based (household) adaptation practices, and (4) local government responses to floods.

Eighteen interviews were conducted in the last three rounds of data collection (see Table 3). Key informants included environmental scientists, agricultural experts, government officials, and farmers. These interviews aimed to explore further their perceptions of the actual transformation of the floodplains as well as adaptation strategies undertaken on the ground. Interview questions focused on: (1) reflections and drivers of changing flood events, (2) development of innovative farming practices, and (3) new ways of adapting to floods. Some key informants consulted in the first round of interviews were re-contacted to provide additional information in the third and fourth rounds. To capture a broader evidence of emerging flood situations concerning agrarian communities’ evolution of adaptation strategies, two farmers living in other deep flooding areas (An Phu and Tri Ton Districts of An Giang Province) were interviewed. Most FGDs and interviews were undertaken face-to-face, except those in the last two rounds. Due to COVID-19 restrictions, these were conducted virtually, with each lasting about one-and-a-half hours.

NVivo software was used to carry out thematic and narrative analyses to delineate communities’ reflections on changing floodscapes and their adaptation responses. We used the grounded theory approach informed by Corbin and Strauss (2015) to implement the thematic analysis. Coding techniques were implemented, including open, axial, and selective coding. These coding processes helped build a hierarchy of emerging themes linked together to present patterns and relationships (Maxwell, 2013). Applying this analytical strategy, we explored the meaning of social memory and how it was connected to agrarian communities’ adaptation strategies in the VMD floodplains over time. Here, we used narrative analysis to capture “event stories” (Esin et al., 2013, p. 205) that emerged from the historical accounts of communities’ adaptation. This approach has been widely adopted to examine human–environment interactions (Bailey et al., 2016), which we found essential to analyse the engagement of agrarian communities in changing environments in this study. We also used content analysis to analyse secondary data sources, including policy documents, books, journal articles, and various newspapers (in English and Vietnamese). These sources were used to understand the transboundary implications of hydropower development, climate change, and changing flood conditions, supporting the analyses of the empirical data collected at the study sites.

## Results and Discussion

### Characterising Transboundary Water Challenges in the VMD Floodplains

We used the two timescales (before and after the 1990s) as our entry points for examining memories of flood conditions in the VMD floodplains. This temporal range corresponds with the onset of the boom in large hydropower dam construction in the Mekong basin (Li et al., 2017; Hecht et al., 2019). Coupled with these developments, climate change, combined with in situ high dyke systems, induces transboundary effects, accentuating flood risks in the VMD (Miller et al., 2020; Pokhrel et al., 2018) (Table 4).

**Table 4:** Transboundary environmental impacts and narratives of changing flood regimes in the VMD floodplains

Time scenarios	Drivers of environmental change in the Mekong region	Narratives of changing flood regimes in the VMD floodplains
Present and future (After the 1990s)	Hydropower development Climate change Expansion of dyked areas for intensive rice production	Transboundary hydrological impacts on the VMD floodplains, characterised by: - Occurrence of abrupt alterations of flood flows ^a^ - Observed delays of flood arrival in the floodplains ^a, b^ - Flood cycles becoming shorter in duration ^c, d^ - Decreased flood frequency in the delta ^e^ - Reduced flood volumes in the delta ^a, f^ - Symptoms of ‘flood-longing *(đợi lũ)*’ and ‘flood-starvation *(đói lũ)*’ ^d, g, h^ Changing flood regimes in unprotected and downstream areas driven by high dyke systems in the delta ^j^ Unpredictable conditions of ‘too much or too little floodwater’ ^a, h, i^
Past (Before the 1990s)	Absence of upstream mainstream hydropower projects ‘Stationary’ climate conditions Minimal water-engineering development in the delta	Unrestrained flows of floods on the Mekong River k Natural cycles of floods (July to December) l Large floods occur almost every five years m Floods retain longer in the delta ^l^

Sources: Adapted from the following sources

^a^Interviews

^b^MRC (2020)

^c^Pokhrel et al. (2018)

^d^Binh et al. (2020)

^e^Park et al. (2020)

^f^Chi (2020)

^g^Le (2019)

^h^Luc (2019)

^i^Truong et al. (2019)

^j^Dang et al. (2016)

^k^Tran (2020)

^l^Le et al. (2007)

^m^Sneddon and Nguyen (2001)

^n^Grumbine et al. (2012)

Such drivers have direct impacts on the ground. They have turned floods from relatively predictable seasonal and cyclical events into unpredictable and disruptive events (Colten & Sumpter, 2009). While large floods previously occurred every five years or so (Le et al., 2007; Sneddon & Nguyen, 2001), since the 1990s, the VMD has witnessed a significant change in flood systems with high unpredictability of frequency and intensity. Flood delays and reductions have become increasingly common (Binh et al., 2020). A prawn farmer in Phu Thanh B (informant recruited in the interview in September 2013) noted that “*We also experienced variations of flood levels more than ten years ago. However, even the low floods we had at that time were much higher than those we have now experienced*” (interview, September 2021). A farmer practising an integrated rice-prawn in a flooding area of An Giang Province shared a similar concern, indicating that “*Similar to previous years, the flood level last year (2020) changed abruptly. It went up and also dropped so quickly*” (interview, September 2021). Explaining the alterations of floodwaters, a senior environmental scientist from Vietnam’s Can Tho University pointed out: “*Now we cannot predict the water flows from the Mekong River because they depend largely on the hydropower dams operating upstream*” (interview, February 2019). Significant flood reductions in the delta recorded in 2015–2016 and 2019–2020 were relevant to hydropower development and El Niño (Lovgren, 2020; MRC, 2020). A senior Mekong ecology expert noted that “*As what I can see in relation to extreme water declines in the delta this year [2019], the main reasons for changing water flows in the floodplains are the El Niño-induced climate, followed by hydropower dams upstream and local dyke systems” (interview, February 2019).* Drawing on their experiential knowledge, observations, and traditional understanding of local flood systems, floodplain residents believed that these abnormal occurrences of floodwater were attributed mainly to human acts. They are considered unethical due to going against “the rules of nature” (Chi, 2020).

Local respondents viewed the alterations of flood regimes in the VMD as negatively impacting their livelihoods. Flood alterations disrupted wild catch fisheries and other natural resources (e.g., aquatic vegetables) and caused difficulties in setting farming schedules. For instance, the farmer of giant freshwater prawns (*Macrobrachium rosenbergii*) in Phu Thanh B (interview, September 2013) observed that: “*Previously, floodwaters could stay in fields for more than two months. Now, I see much evidence of delayed floods. This causes challenges for us to schedule prawn crops.*” Having a similar view, a researcher in Dong Thap Province added, "*Delayed floods do not help much in reality, as they disturb the growth cycles of most aquatic species.*” (quoted in Luc Tung on Lao Dong News, 2019). As the next section will elucidate, farmers’ adaptation practices reveal their efforts to adjust to the increasingly irregular flood rhythms of the VMD, highlighting how social memories shaped their practices.

### Social Memory and Community Connectedness to the Changing Floodscapes

Social memory forms cognitive and emotional connections between environmental impacts and agrarian communities’ attitudes and behaviors towards environmental change. Historical narratives of rural societies’ adaptation are embedded in particular spaces and cultures that inform their interactions with floods and shape their behaviors and responses. In the VMD, social memories of past flood experiences feed into learning processes to deal with challenges over time (from large floods to little flood events). The prawn farmer in Phu Thanh B noted: “*I observe, learn, and accommodate my farming practices to local flood conditions. Seeing that my earlier giant freshwater prawn does not bring any profits due to flood disruptions, I realise this is time for me to change*” (interview, September 2021). Social memory, in this sense, serves as a record of changing flood events that inform farmers' livelihood changes. As Barthel et al. (2010) noted, social memory serves as a carrier of knowledge, experience and practice in working through human interactions with nature.

**Fig. 3:**
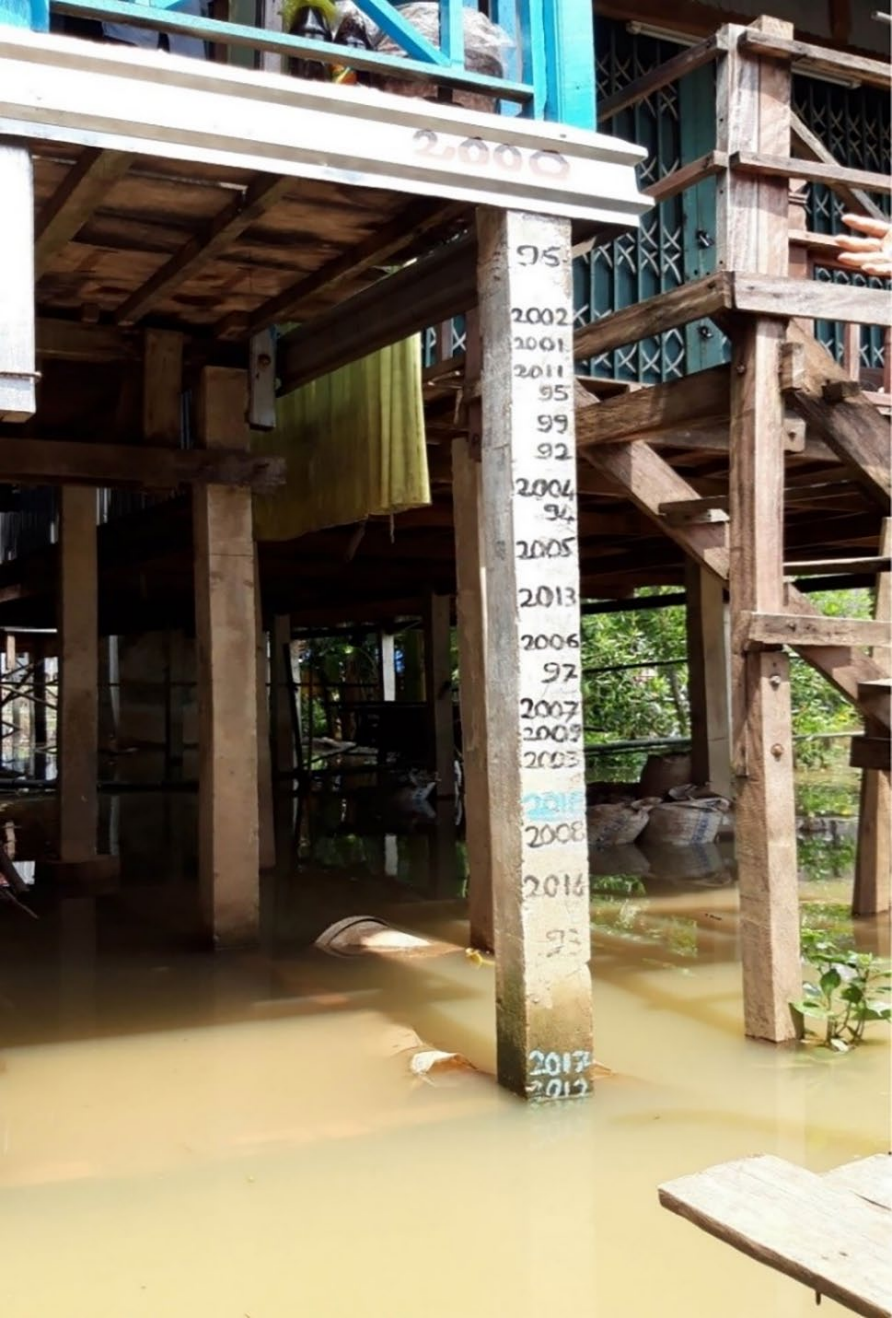
Flood reminiscences are demonstrated by the reduction of flood levels recorded in a rural flood-prone community of An Giang Province (Source: Phong Thanh Le, September 2019)

Flood reminiscences provoked by agrarian communities were demonstrated by expressions of “flood-longing” *(đợi lũ*) (Le, 2019) as well as concerns about “flood starvation” *(đói lũ*) when seasonal floods do not come as expected (Fig. 3). The prawn farmer in Phu Thanh B shared the similar observation of local flood conditions to the one when the first interview was conducted (September 2013), saying that: “*we have experienced recurring delays and sporadic returns of large floods in recent years. Often, floodwater flows over riverbanks in July. However, I have not seen any sign of that thus far. Recalling it from fifteen years ago, floodwaters would have filled up my prawn-cultured farm by now*” (interview, September 2021). The prawn farmer’s observation echoed the former leader of An Giang Province: "*Those who live in the VMD have learnt by heart this common saying: ‘Floodwaters jump over riverbanks in July*^1^*’, but I have not seen this natural phenomenon in recent years.*” (Quoc & Manh, 2019). These narratives resonate with the feelings about flood disruptions shared by many floodplain community residents, as reported by local newspapers (Cuu, 2019; Hong & Thanh, 2019; Le Xuan, 2019).

In the VMD floodplains, memories of “beautiful floods” *(lũ đẹp)* (Ehlert, 2012) have, however, been fading and replaced by experiences of flood precarity and disruptions, making it “flood seasons full of sadness” (*mùa lũ buồn*) (Quoc et al., 2019). As the prawn farmer in Phu Thanh B noted: “*living on this flood-based occupation, I am always happy to see the regular returns of floods, especially major ones, as they provide good opportunities for my farming*” (interview, September 2021). Sharing the same feeling, a farmer cultivating floating rice in An Giang noted that “*my floating rice is growing high now. However, there is no sign of floods that could come and fill up the rice field. I am feeling so anxious about it and so much longing for floods*” (interview, September 2021). These narratives suggest agrarian communities’ mixed perceptions of floods, ranging from their appreciation of flood benefits in support of rural livelihoods to growing anxieties about flood uncertainties in the present and future.

Social memory serves as a conduit for the transfer of intergenerational knowledge leading to the social construction of learning communities of practice. In this study, reflections on past environmental experiences give farmers and their communities the capacity to learn to adapt (Tran, 2020). The prawn farmer in Phu Thanh B commented that “*under the constraining conditions of floodwater, it is not possible to keep cultivating giant freshwater prawn. So I decided to switch to the white-leg shrimp farming, which provides better economic returns. I have practised this over the past five years, and my son is now continuing my work. Many neighouring farmers have followed me and helped expand this farming across the commune*” (interview, September 2021). Connecting this narrative to the work of Valencia et al. (2019), the contention here is that the VMD floodplains are “reservoirs of social memory” (p. 1471) that nurture agrarian communities’ aspirations and motivations in dealing with natural environments (Taylor, 2001) and shape communities’ adaptive behavior and actions to overcome environmental challenges over time. In light of social memory, the paper suggested that the ecological and experiential knowledge accumulated from agrarian communities’ “living with floods” (Liao, 2019) in the past would help (re)construct adaptation strategies in dealing with the extreme conditions of too much or too little floodwater (Truong et al., 2019; Tung, 2019). Social memory, therefore, contributes to strengthening community-based (re)connections to nature that, in return, inform the new perceptions and knowledge of emerging environmental conditions and help build their capacities to learn and act upon change (Ives et al., 2018).

Social memory highlights the profound imprints of nature (floods) on agrarian communities, illustrating emotional bonding and strong connections between them. Our data suggested that, unlike out-migrants (primarily young people) who are identified as not long-term “friends with the water” (Merten et al., 2021: 60), those who decide to stay back (mostly elderly people) are likely to hold a more profound sense of belonging to floods. Of course, such narratives risk perpetuating severe problems associated with romanticising rural and agrarian communities, including uncritically imposing moral claims onto people living in precarious situations or forced into unsustainable livelihood transitions. Somewhat differently, the farmer cultivating floating rice in An Giang expressed that “*my family has long lived in this area and cultivated this type of farming… Despite experiencing low floods, we have to stick to it*” (interview, September 2021). This characterises a so-called “moral identity” held by floodplain communities (Clarke & Mayer, 2017, p. 137), who do not betray the natural (flood) endowments offered to them. Relating this to the context of the VMD floodplains, we suggest that flood reminiscences help provoke communities’ expressions of a sense of belonging and strong connections to floods (Davenport & Anderson, 2005); they offer a means of survival and sustain the emotional well-being for communities in dealing with environmental challenges.

### Narratives of Adaptation Shifts

Social memory provides the capacity for change in cases where people have sufficient resources to act on their shared perceptions of altered circumstances. In such cases, collectivised narratives of past community-flood interactions can be harnessed to enhance community capacity. In this study, social memory provides a means through which agrarian communities have used their experiential knowledge produced through their lifetime interactions with flood environments. This involves a gradual shift from incremental to transformative adaptation.

Incremental adaptation revolves around traditional adaptive pathways, which are locally focused and confined to small-scale (district and community level) geographies of place-based resource livelihoods. The adaptation narrative evidenced this by a small group of farmers in An Giang Province in the late 1970s: they built low dykes to prevent floods into their fields and protect rice crops. This was subsequently formalised into the local adaptation policy and widely practised in the local communities and beyond (Howie, 2011). In this study, incremental adaptation was primarily demonstrated through how agrarian communities (mostly poor farmers) exploited resources from seasonal floods to support their livelihoods. This form of adaptation was illustrated in FGDs with local farmers:

We depend largely on wild fish capture and collection of other aquatic resources for our livelihoods in the flood season. During this period of time, we can only make our living by catching fish rather than doing anything else. (FGD, Phu Thanh B, January 2014).

My family earned a living by capturing wild fish and collecting aquatic vegetables. Floods provide good income for us, and we appreciate it a lot. (FGD, Thoi Hung, November 2013).

Our data suggested that shared memories of experiencing floods led to a shared understanding of how to adapt local livelihoods to new environmental conditions better. This indicates evidence of social learning that emerged from historical experiences in engaging with floods (Dickson-Hoyle et al., 2020). In Thoi Hung, for example, the collective memories of flood challenges between the local government and farmers in their early-day settlements in the commune contributed to an improved understanding of how local farming production activities would be reorganised by harnessing the advantages of floods. This led to the government’s rectification of the closed irrigation scheme that allowed for the free-flowing intake of floods into farmers’ fields (Tran & Rodela, 2019). From the social memory perspective, this collaborative form of learning is vital for building community resilience.

### Moving Towards Resilience

Social memory triggers transformative adaptation by setting the contexts for collective understandings of emerging water challenges facing the floodplains. Drawing from social memories as indicated by agrarian communities’ experiences in dealing with real-life situations, provincial authorities in An Giang shifted to more sustainable agricultural production methods, promoting non-intensive rice farming practices (An Giang People’s Committee, 2014). This was aligned with Resolution No. 120/NQ-CP on the VMD’s resilient development plan in responding to emerging environmental conditions in the VMD floodplains (Vietnamese Government, 2017; Government Prime Minister, 2019). From the social memory perspective, this policy shift indicates an institutional capacity to learn from the past and harness strategies to deal with the future (Dickson-Hoyle et al., 2020). This approach helps promote the collective forms of environmental stewardship (Bennett et al., 2018) to enhance community resilience. A senior Mekong expert observed that:

For my last visit to Dong Thap and An Giang, I realise that farmers are changing their farming systems. In Dong Thap, farmers have filled up their fields with floodwater, while farmers in An Giang cut down rice crops from 3 years 8 crops to 2 years 5 crops (Interview, November 2020).

Efforts to move towards the evolutionary resilience approach were particularly well reflected at the community level. With lessons learned from their social memories, agrarian communities reorganised their farming practices towards ecologically-based farming systems. In An Giang Province, for instance, farmers shifted away from quantity-based (i.e. multiple rice crop farming systems) towards quality-based (e.g., high-quality rice varieties) production modes or diversified into cash crops to earn additional income (Nguyen et al., 2020; Vo et al., 2019). A senior official from An Giang Irrigation Agency noted: “*Ecosystem-based adaptation should be promoted in deep inundation areas in the province by allowing floods to enter fields to replenish sediment and nurture wild fisheries.*” (Interview, December 2013). Farmers shifted to alternating farming systems in high flooding areas, such as rice-prawn, to improve household income. A farmer practising this model in An Phu District noted, "*I started the rice-prawn farming system in 2017, seeing that I can harness floodwater to culture prawn after harvesting the summer-autumn rice crop. The reproductive rice (luá chét) and rotten rice stem provide good sources of feed for prawn*” (interview, September 2021). Given the VMD's current water challenges as either too much or too little floodwater, looking forward, learning to live with environmental changes and uncertainty would be an essential approach to building resilient agrarian communities (Kuang & Liao, 2020; Marschke & Berkes, 2006).

Flood-resilient strategies imply agrarian communities’ increased recognition of the essential role of natural processes they engage with. In light of the evolutionary resilience approach, community reminiscences of floods through their inherent connections characterise a unique culture of resilient agrarian communities in the VMD (Clarke & Mayer, 2017). Here, flood reminiscences present the essential values of human-flood interactions embedded in agrarian communities’ everyday adaptation practices. It empowers them to deal with environmental change (i.e., too much or too little floodwater) by translating local ecological knowledge and experiences into actions to enhance resilience, assuming they have sufficient social capital and material resources to initiate adaptive strategies.

## Conclusions

This paper suggests that the altered water conditions in the VMD floodscapes shape how agrarian communities collectively remember, value, and make sense of floods. In contributing a nuanced understanding of social memory, we have shown its potential value as a learning connector that can link reflections of past community engagements with floods to present circumstances and future water challenges. When coupled with sufficient resources to facilitate adaptive capacity-building, social memory can thus catalyse collective forms of resilience when communities transition from incremental to transformative adaptation.

On-the-ground realities reveal that “incremental” and “transformative” adaptation practices are intertwined. However, local governments promote only the latter to enhance community capacities in adapting to the Mekong’s changing flood conditions. These thematic areas need to be further expanded in the broader social, environmental, and institutional contexts of the Global South, which are highly vulnerable to coupled climate-development challenges. Future research needs to examine how social memory shapes agrarian communities’ adaptive capacity while dealing with these complex challenges.

The Vietnamese government has successfully turned the VMD into a productive agrarian space. In doing so, we have argued that various environmental and social problems that have become evident are often only visible locally. At the same time, transboundary drivers remained hidden from sight and omitted mainly from formal planning processes. Agrarian communities, while building on their experiential knowledge in the conventional context of living with floods, have taken proactive steps towards working within the transformative agenda in the new challenging water context of the VMD. This demands communities to learn continuously, self-organise, and co-evolve to adapt to change. Notably, the collective reminiscences of changing water regimes help foster community-flood (re)connections that serve as a critical foundation for enhancing community resilience.

## Data Availability

The data that support the findings of this study are available on request. The data are not publicly available as they contain information that could compromise research participant privacy/consent.

## Footnotes

1 This remark indicates the experiential knowledge of rural societies on the regular event of peaking floodwater that occurs in July (Lunar month calendar).
